# Replicative Stress and the FHIT Gene: Roles in Tumor Suppression, Genome Stability and Prevention of Carcinogenesis

**DOI:** 10.3390/cancers6021208

**Published:** 2014-06-04

**Authors:** Jenna R. Karras, Carolyn A. Paisie, Kay Huebner

**Affiliations:** Department of Molecular Virology, Immunology and Medical Genetics, The Ohio State University Wexner Medical Center, Columbus, OH 43210, USA; E-Mails: jenna.karras@osumc.edu (J.R.K.); Carolyn.paisie@osumc.edu (C.A.P.)

**Keywords:** oxidative stress, reactive oxygen species, common fragile sites, genome instability

## Abstract

The fragile *FHIT* gene, encompassing the chromosomal fragile site FRA3B, is an early target of DNA damage in precancerous cells. While vulnerable to DNA damage itself, FHIT protein expression is essential to protect from DNA damage-induced cancer initiation and progression by modulating genome stability, oxidative stress and levels of accumulating DNA damage. Thus, FHIT, whose expression is lost or reduced in many human cancers, is a tumor suppressor and genome caretaker whose loss initiates genome instability in preneoplastic lesions. Ongoing studies are seeking more detailed understanding of the role of FHIT in the cellular response to oxidative damage. This review discusses the relationship between FHIT, reactive oxygen species production, and DNA damage in the context of cancer initiation and progression.

## 1. Introduction

Genomic instability is a hallmark of human neoplasia, present in varying degrees in all stages of cancer, from precancerous to advanced cancer. Instability in the form of chromosomal instability occurs in nearly 90% of human malignancies as structural and numerical anomalies [1,2]. Defects in DNA replication, aberrant checkpoint responses, and oxidative stress contribute to the expansion of instability throughout the genome during neoplastic progression. Common fragile sites (CFSs) are specific, conserved chromosomal regions that are highly sensitive to replication stress and are involved in the development of cancer. Under conditions that impair DNA replication, fragile sites show breaks, gaps and rearrangements in a variety of normal and cancer cells. The *FHIT* gene is positioned at one of the most active CFSs, FRA3B, and is one of the most frequently altered genes in preneoplasia and cancer [3]. Alterations at this locus include deletions, translocations and promoter methylation, often resulting in the loss or reduction of expression of the FHIT protein. Absence of one *FHIT* allele can occur in normal tissues and lead to areas of metaplasia with reduced FHIT expression. Loss of the second *FHIT* allele can lead to complete loss of FHIT expression, which is observed in many dysplastic lesions [4]. Homozygous deletions leading to total loss of specific FHIT exons, and thus total loss of FHIT protein, has been demonstrated in primary cancers such as esophageal, gastric and lung carcinomas [3]. While the FRA3B locus is highly vulnerable to DNA damage due to replication stress, the FHIT protein, paradoxically, is a tumor suppressor and genome caretaker that modulates genome stability, oxidative stress and level of DNA damage that accumulates beginning in precancerous lesions [5,6,7,8,9,10,11,12]. The instability at FRA3B and the subsequent loss of FHIT protein expression is detected in precancerous cells and precedes the instability observed at other genomic loci. Nevertheless, more than fifteen years after the identification of the *FHIT* gene, encompassing the FRA3B locus, its role as “guardian of the preneoplastic genome” is not widely known and questions remain regarding the role of the loss of FHIT in the cellular response to oxidative damage [13]. In this review, we will discuss the ability of the FHIT protein to participate in the response to oxidative damage, and the implications for neoplastic progression.

## 2. Oxidative Stress and the Mitochondrial Fraction of FHIT Protein

**Figure 1:**
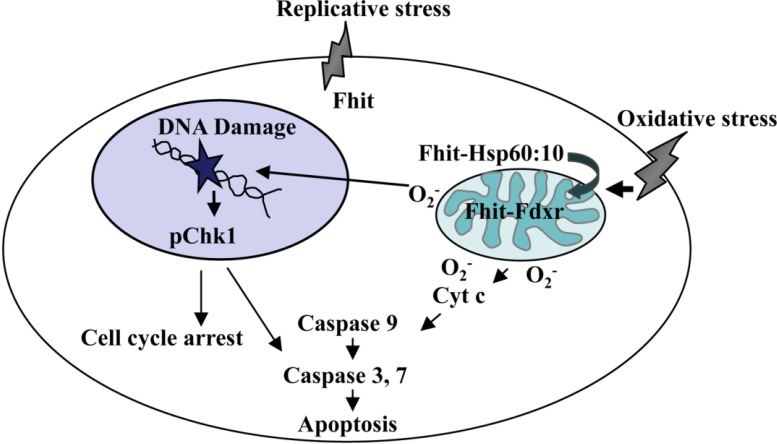
FHIT: suppressor and caretaker. In response to oxidative stress, FHIT protein localizes to the mitochondria via Hsp complex where it interacts with and stabilizes ferredoxin reductase, leading to enhanced production of reactive oxygen species, stimulation of cytochrome c release and subsequent activation of the Caspase cascade under conditions of severe oxidative stress. In response to genotoxic stress, FHIT participates in the checkpoint response to DNA damage via Chk1 to commit cells to cell cycle arrest and, if DNA damage is extensive, to apoptosis. Importantly, FHIT-deficient cells are resistant to oxidative and genotoxic agents and develop preneoplastic changes.

The FHIT gene was characterized as a cancer suppressor soon after it was cloned in 1996, though there was not universal agreement concerning its suppressor role [14,15,16]. FHIT knockout mice display a moderately increased frequency of spontaneous tumors and greatly increased susceptibility to carcinogen-induced tumors compared to wild type mice of the same strain [17,18]. Furthermore, viral-mediated FHIT gene therapy inhibits tumor development and induces caspase-dependent apoptosis [17,18,19,20]. Interest in other proteins that interact with FHIT and mediate apoptotic pathways supporting its tumor suppressor function led to the discovery of FHIT involvement in the response to oxidative stress. Through protein co-immunoprecipitation studies Trapasso *et al.* showed that FHIT interacts with Hsp60 and ferredoxin reductase in cells overexpressing FHIT [9]. Hsp60 is a molecular chaperone that complexes with Hsp10 and is important for proper folding, stability and import of proteins into the mitochondria. Knockdown studies of this complex result in decreased levels of FHIT in the mitochondria suggesting this complex is important for the stability of FHIT and/or for its import into the mitochondria [9]. Ferredoxin reductase, Fdxr, is a mitochondrial flavoprotein transactivated by p53 that is responsible for shuttling electrons through the electron transport chain to mediate intracellular oxidants and regulators of apoptosis. Overexpression of FDXR increases reactive oxygen species (ROS) production activating apoptosis in tumor cells exposed to H_2_O_2_. The interaction between FHIT and FDXR suggested that ROS production contributes to FHIT-induced apoptosis. Indeed, cells overexpressing FHIT produced significantly elevated levels of ROS after H_2_O_2_ treatment, and also exhibited elevated FDXR protein levels in mitochondria. This FDXR stability is due to protection from proteasomal degradation by FHIT and implies that FHIT-induced ROS production is dependent on FDXR level [9]. Further investigation led by Rimessi *et al.* demonstrated that FHIT increases calcium uptake into the mitochondria by sensitizing the low-affinity Ca^2+^ transporters in both intact and permeabilized cells [21]. Accumulation of calcium in the mitochondrion is important for initiating the morphological changes observed in apoptosis. Consistent with previous reports, Pichiorri *et al.* also noted that FHIT-deficient cells avoid G2/M cell cycle arrest and apoptosis [22]. Thus, in response to oxidative stress, FHIT is imported into the mitochondria where it interacts with FDXR, thereby increasing FDXR protein levels to enhance intracellular ROS production (see Figure 1) and mitochondria calcium uptake, triggering apoptosis. Conversely, cancer cells deficient of FHIT expression cannot protect FDXR from proteasomal degradation and escape apoptosis as they are less sensitive to oxidative stress. Therefore FHIT-deficient preneoplastic cells survive carrying low levels of oxidative DNA damage that may contribute to increased mutation burden and ultimately neoplasia.

## 3. The FHIT-Substrate Complex

**Figure 2:**
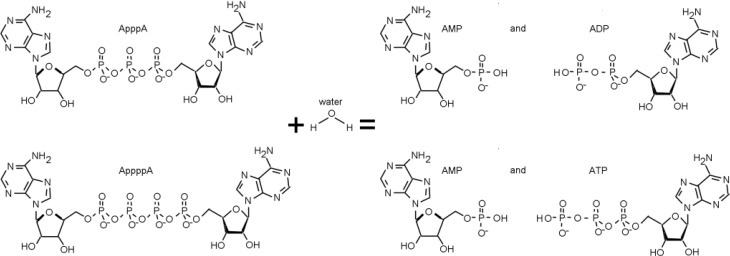
The FHIT substrates and hydrolysis reaction. Diadenosine triphosphate and diadenosine tetraphosphate are hydrolyzed to AMP + ADP and AMP + ATP, respectively, by wild type FHIT.

FHIT is a member of the histidine triad (HIT) superfamily of enzymes responsible for the hydrolysis of diadenosine polyphosphate molecules, though a biological function for this hydrolytic activity is not known. The preferred substrate of FHIT is Ap_3_A but FHIT can also hydrolyze Ap_4_A, though the actual *in vivo* substrate for FHIT has not been directly identified (see Figure 2). Each His amino acid of the Histidine triad of the *FHIT* gene has been mutated and ability of mutants to cleave the Ap_3_A substrate *in vitro* was assessed, as were mutations in other significant amino acids, identified by examining the crystal structure of the protein [23,24].

**Figure 3:**
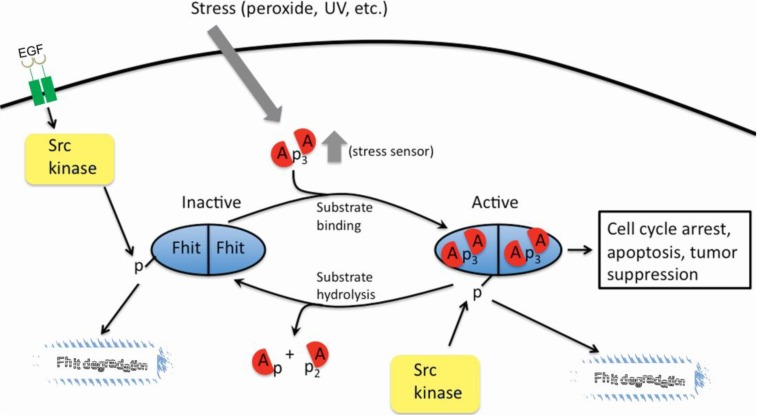
FHIT-substrate complex as a signaling molecule. Intracellular Ap_3_A substrates may increase following external stimuli such as peroxide and UV light. The FHIT signaling pathway is activated when FHIT binds Ap_3_A substrate. Hydrolysis of Ap_3_A substrate into AMP and ADP, or Src phosphorylation of Tyrosine 114 and subsequent degradation may inactivate the signaling pathway.

In early studies it was shown that mutation of the central H residue of the histidine triad to N allowed tight binding of the FHIT-substrate complex but abrogated cleavage of the substrate [23]. This mutant, H96N, was nearly as good at tumor suppression as was the wild type protein, suggesting the hypothesis that the FHIT-substrate complex sends the tumor suppression signal [6]. A number of other mutants did not cause cell apoptosis when overexpressed in cancer cells and thus did not retain suppressor activity [25]. These mutants, H96N as well as mutants that did not retain substrate binding activity were used in more recent experiments to assess the level of their binding to Hsp60/Hsp10 and to show that the H96N mutant but not the non substrate-binding mutants, was able to bind the Hsp chaperone complex, enter mitochondria, bind the FDXR protein and thereby participate in increasing the production of ROS [9,22]. Thus, the catalytically dead mutant that binds substrate well, FHIT H96N, displays more extensive oxidative damage, activation of apoptosis and suppression of tumors, confirming that enzymatic activity is not required for its tumor suppressive function. FHIT is also a target of tyrosine phosphorylation, specifically residue 114, by Src family protein kinases both *in vitro* and *in vivo* [26]. This residue is located in a twenty amino-acid unstructured loop region of FHIT. However, phosphoFhit is not detected in the mitochondria, most likely due to rapid degradation and lack of Hsp60 interaction, and is apparently not a participant in tumor suppressive functions. It has been reported that phosphorylation of FHIT is a signal for down-regulation of FHIT expression through degradation, sent by activation of the EGFR-Src signal pathway [27]. Site-directed mutagenesis of Tyrosine 114 has provided evidence for the importance of this residue in substrate binding and has been shown to directly affect the stability of FDXR when this interaction occurs. Interestingly, mutations at this position give the most dramatic effect on the structural function of the loop region by perhaps partial or complete abrogation of the binding site preventing substrate binding and subsequent suppressor signaling (see Figure 3). The authors conclude that tyrosine 114 is critical for the formation of the FHIT-substrate complex and subsequent tumor suppressor signaling via ROS induction and activation of Caspase-dependent apoptosis.

## 4. FHIT and Genome Stability

In continuing studies of the role of FHIT in DNA damage and response pathways, Saldivar *et al.* revealed the genome caretaker function of FHIT [8]. To define the role of FHIT in protecting genome integrity, Saldivar *et al.* examined levels of spontaneous DNA damage upon loss of FHIT protein [28]. In normal, transformed and cancerous cell types, decreased expression of FHIT resulted in increased levels of spontaneous DNA double-strand breaks (DSBs) and replication fork stalling. Analysis of nucleotide pools revealed that dTTP levels were significantly reduced upon loss of FHIT protein expression and thus unable to support efficient DNA replication. Importantly, the DNA damage generated from the replication defects in FHIT-deficient cells did not cause activation of Chk1 and the S-phase cell cycle checkpoint response. Accordingly, spontaneous DNA damage induced by loss of FHIT is transmitted to daughter cells leading to genome instability. FHIT loss-induced replication stress eventually leads to chromosomal instability as was observed by the formation of micronuclei and aneuploidy. Thus, FHIT, which is reduced in expression in most human cancers, is a genome “caretaker” whose loss initiates genome instability in preneoplastic lesions.

**Figure 4:**
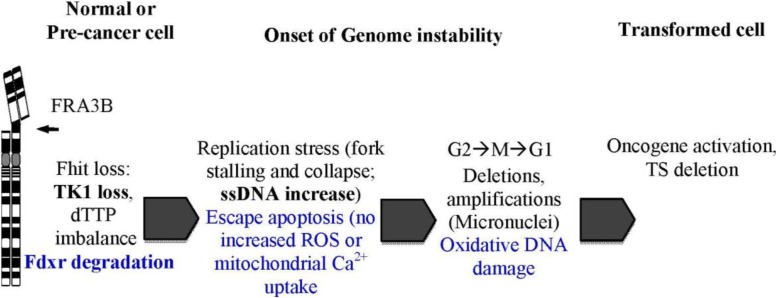
Model for FHIT loss-induced genome instability and neoplasia formation. Deletions in *FHIT* alleles occur due to FRA3B fragility. FHIT loss causes TK1 loss and dTTP pool insufficiency, triggering stress-induced increases in ssDNA. FDXR no longer protected from proteasomal degradation in absence of FHIT allowing escape from apoptosis as cells are less sensitive to oxidative stress. Together, replication stress and evasion of apoptosis leads to accumulation of genomic changes. FHIT loss-induced genome instability thus increases the likelihood of activating mutations in oncogenes and inactivation of tumor suppressor genes (TS); selective pressures allow clonal expansion.

Balanced deoxyribonucleotide triphosphate (dNTP) pool levels are essential for efficient DNA synthesis and for the maintenance of genome stability. The depletion of dNTP pools can affect both DNA replication and genome stability [8]. Thymidine kinase 1 (TK1), an enzyme that catalyzes the ATP-dependent phosphorylation of thymidine to thymidine monophosphate, is involved in the synthesis of dTTP via the scavenger pathway [8,29]. Expression of TK1 is high during S and G2 phase to ensure sufficient dTTP production for efficient DNA synthesis [8]. It was previously shown that loss of FHIT results in a decrease in TK1 expression, resulting in a reduction of dTTP [8]. While this reduction results in a level of dTTP that affects DNA synthesis, the effect is not severe enough to activate Chk1 and block cell cycle progression (see Figure 4) [8]. Further research will be necessary to understand how FHIT, TK1, and dTTP pools may contribute to the generation of genome instability, including an expected role in the generation of the mutations observed in FHIT knockout mouse exome sequences.

## 5. Oxidative Stress, ROS, and FHIT

Previous research has demonstrated a relationship between oxidative stress, ROS, and FHIT that ultimately results in the disruption of various cellular processes [13,30]. As discussed above, oxidative stress and ROS can negatively impact DNA and results in mutations and/or lesions that require repair to maintain DNA integrity. Previous studies have demonstrated that FHIT: (1) can increase ROS production due to oxidative stress via its interaction with FDXR in the mitochondria (see Figure 1) and (2) at normal levels in normal cells may help to protect DNA from damage caused by endogenous ROS [28]. It is possible that the instability generated by FHIT loss, in combination with increased ROS production due to endogenous or exogenous exposures, leads to disruptions in normal cellular functions that ultimately generate DNA point mutations. Additionally, FHIT can act as a modulator of the stress response and may affect the stability of proteins involved in cell-cycle block, apoptosis, or checkpoint activation [13]. Under stressful conditions, cells may accumulate such severe DNA damage that apoptosis is necessary to prevent the passage of DNA mutations that may accumulate in damaged cells to future generations. Previous studies of the role of FHIT in ROS production have employed cells with over-expression or absence of FHIT; thus understanding of the physiological role of normal levels of the FHIT-FDXR complex and its role in fine-tuning the response to oxidative stress will require further research.

## 6. ROS, Mutations and FHIT

A number of different classes of mutations can be generated by ROS. The signature mutations generated by ROS are CC>TT substitutions that occur in tumors in the absence of ultraviolet (UV) exposure, but the most common mutation that results from oxidative damage is oxidation of guanine to form 8-oxo-2' deoxyguanosine (8-oxoG) [31]. Copying of 8-oxoG during repair or replication results in C>A (G>T) mutations while C>T (G>A) and C>G (G>C) mutations can be generated as a result of the formation of 8-oxoG adducts [31,32,33]. T>C mutations in hepatocellular carcinomas have been shown to result from the addition of bulky DNA adducts to adenine [33]. Cytosine glycol and thymine glycol can be generated as a result of ROS-induced mutagenesis; thymine glycol and deamination of adenosine or cytidine can induce A>G, G>A, C>T, and T>C mutations [31,32]. Somatic single base substitutions, that are sequence context-dependent, may be generated by DNA oxidation resulting from electron transfer; more specifically, guanine and 8-oxoG can undergo ROS-dependent, and sequence context-dependent, oxidation reactions [33]. Thus, it is possible that mutations observed in DNAs of FHIT-deficient cells and tissues are generated due to oxidative damage generated by ROS.

In addition to a number of specific mutations that are the direct result of ROS-induced mutagenesis, loss of FHIT expression can promote expansion of FHIT-deficient cells, in selective conditions, and accompanying mutations [10]. The mutations acquired by cells are important for the development of numerous phenotypes necessary for subsequent tumor development; FHIT-deficient cells, being genomically unstable, are more likely to acquire the mutations necessary for cancer development [8,10,34]. Alterations in FHIT-deficient cells include changes in proliferation as well as changes in apoptosis and survival pathways [10]. Thus it is expected that there may be a number of different causes of mutations, and various classes of mutations, in FHIT-deficient cells. Through *in vitro* studies of mouse embryo fibroblasts (MEFs) and kidney cell lines from FHIT knockout mice, it was demonstrated that mutations occur that provide selective survival and proliferative benefits for FHIT-deficient cells [10]. Increased levels of chromosome copy number variations (CNVs) and point mutations were reported in FHIT knockout tissues and derived cells by CNV and exome sequence analysis [10]. It is possible that oxidative stress, in combination with FHIT loss, creates an environment in which there is selection of cells that are capable of surviving under these conditions. In addition, FHIT-deficient cells, in the presence of oxidative stress, can accumulate mutations in p53, which results in its loss or overexpression of mutant protein and decreased expression of downstream apoptosis and DNA damage response pathways.

Defective DNA repair mechanism(s), such as base excision repair (BER) or mismatch repair (MMR), may also have a role in the generation of mutations as a consequence of oxidative DNA damage and/or oxidative stress. BER involves the removal of a single lesion by a DNA glycosylase, followed by generation of an apurinic or apyrimidinic (abasic or AP) site [31,35]. Initiation of BER has previously been shown to occur following the generation of oxidized DNA base mutations [31]. MMR functions to repair nucleotide mismatches via detection and removal of the mismatched base followed by synthesis by DNA polymerase to correct the error [36]. The repair of such lesions may result in the generation of C>T mutations [37]. Both BER and MMR can be activated by the presence of uracil(s) that may be generated by cytosine deamination and previous research has shown that oxidative damage can result in the deamination of cytosine and generate C>T mutations [38,39]. As discussed above, FHIT-deficient cells have decreased availability of dTTP and it is possible that this may have a role in incorrect repair at an AP site such that an alternate base, in place of thymidine, is inserted [8].

## 7. Discussion

As noted above, the *FHIT* gene encodes a protein that is both a tumor suppressor and a genome caretaker and has a role in various processes, including production of and response to oxidative damage, that are involved in the generation of precancerous lesions [8]. As a result of oxidative stress, FHIT may be imported into the mitochondria as part of a complex where it interacts with FDXR and enhances intracellular ROS production. The balance between ROS, FHIT, and oxidative stress is crucial as a disruption in balance of these factors ultimately leads to the disruption of a number of cellular processes and likely results in an increase in numbers of mutations [13,30]. It is possible that MMR and/or BER may play a role in the repair of such mutations. In summary, there is a complex relationship between FHIT expression, ROS, and oxidative stress that plays a role in protection from or susceptibility to mutations and tumor development. When FHIT is expressed at physiological levels, there is likely a balance involving the FHIT-FDXR interaction that finely tunes the level of ROS production and oxidative stress. It is likely a disruption of this balance that results in mild oxidative DNA damage and in an increase in mutations when there is decreased FHIT expression. Therefore, the level of FHIT expression is crucial for the response to replicative and oxidative stresses, demonstrating the complex nature of the functions of FHIT. A recent large scale study of available cancer sequencing data reported identification of multiple mutation signatures in 30 types of cancer, and certain mutation signatures have been linked to probable causes such as age, smoking, BRCA1/2 mutations, or MMR deficiency [40]. With the amount of sequencing data available in publicly accessible databases, as well as an increase in sequencing of tumors, it may soon be possible to identify, based on the pattern and type of mutations, a specific FHIT-ROS mutation signature for cancers in which there is a change in the balance between the FHIT-FDXR complex and ROS production. Studies investigating the relationship between FHIT and ROS have also elucidated the connections between various pathways that are known to be altered in FHIT-deficient cells such as those for apoptosis, ROS production, and DNA damage response checkpoint activation. Crucial questions remain regarding the normal physiological roles of FHIT in mitochondrial ROS production and calcium uptake.

Although previous studies determined that loss of FHIT leads to DNA damage, followed by progressive genome instability, the DNA repair mechanism(s) employed by FHIT-deficient cells carrying DNA damage remains elusive. With the increase in sequencing of tumors, as described above, it may be possible to determine what kinds of mutations or mutation signature(s) occur in FHIT–deficient cells and tissues, with and without carcinogen exposure, in both early and late stages of transformation. The types of mutations seen in these FHIT-deficient cells may provide insight into types of repair used in FHIT-deficient cells.

Future research efforts will define in more detail the signal pathways affected by FHIT loss, both pathways involved in early steps of neoplastic development as well as repair pathways. Future sequencing efforts aimed at complete definition of sequence and expression alterations in selectively expanding FHIT-deficient preneoplastic and tumorigenic clones will define the various transformation pathways exploited during selective growth of the deficient cells on the way to cancer development. A main goal of this research will be to define early changes in FHIT-deficient preneoplastic cells that will provide targets to prevent progression to neoplasia.

## 8. Conclusions

While the FRA3B locus is highly vulnerable to DNA damage due to replication stress, the FHIT protein, paradoxically, is a tumor suppressor and genome caretaker that modulates genome stability, oxidative stress and level of DNA damage that accumulates beginning in precancerous lesions. Studies have shown the importance of the Fhit-substrate complex, as opposed to its catalytic activity, in promoting the tumor suppression signal. However, upon loss of Fhit expression, this suppression signal is lost allowing the accumulation of DNA damage due to the inherent replication stress and increased levels of ROS that are generated. The combined effects of replication stress and ROS production contributes to the increased numbers of C>T and T>C mutations that are observed in Fhit-deficient cells. Future research will provide insight into the mechanisms of Fhit-loss induced genome instability and will further characterize the types of mutations due to Fhit loss that mediate the progression from preneoplasia to cancer.
